# A comparison of assisted human reproduction (AHR) regulation in Ireland with other developed countries

**DOI:** 10.1186/s12978-022-01359-0

**Published:** 2022-03-05

**Authors:** Olivia McDermott, Lauraine Ronan, Mary Butler

**Affiliations:** 1 College of Science and Engineering, National University of Ireland, Galway, Ireland; 2 College of Science, Institute of Technology, Sligo, Ireland

**Keywords:** Assisted human reproduction, IVF regulation, Ireland, In vitro fertilisation

## Abstract

**Background:**

Assisted human reproduction (AHR) treatment is not regulated in Ireland although it has been practiced since 1987. Thus, Ireland is one of the only European countries without any form of AHR specific regulation. This literature review research aimed to provide a comprehensive and comparative overview of AHR regulation and any associated literature to compare Ireland and other developed countries.

**Methods:**

Systematic searches were conducted in several databases (Google Scholar, Web of Science, MEDLINE, SCOPUS and official government websites) utilising search strings in relation to AHR legislation for each country under review. A final review of 155 research articles were eligible after screening related to legislation in each country for inclusion. The findings were synthesised and summarised by legislation in each country.

**Results:**

Different countries offer different levels of ART and IVF provision and services in terms of the type of services allowed, financial support, age, sex and eligibility of recipients. The UK’s oversight legislation combined with the Netherlands financial legislation section provides as being most effective hybrid model of best practice for adoption in Ireland.

**Conclusions:**

This research concluded that there is no AHR legislation in any country that can be described as all-encompassing in terms of the services allowed, financial support and age of recipients. It was concluded that significant changes need to be made to the Irish draft legislation which is in limbo with the government for the last 3 years in order to meet Irish patient needs.

## Plain language summary

Ireland is one of the only European countries without any form of assisted human reproduction specific regulation. This research aimed to review of assisted human reproduction regulations to compare Ireland with other developed countries. There is no assisted reproduction legislation in any country that can be described as perfect. The UK’s legislation combined with the Netherlands financial legislation section is concluded as being most effective hybrid model of best practice for adoption in Ireland. It was concluded that significant changes need to be made to the Irish draft legislation which is in limbo with the government for the last 3 years.

## Background

### Introduction

Assisted human reproduction (AHR) is one of the fastest-growing healthcare industries in recent years, with in-vitro fertilisation (IVF) experiencing the most significant increase in demand. On average, there are 6000 cycles of IVF treatment completed in Ireland each year [1]. The figures now show that one in four couples will experience infertility issues, and whilst all might not need IVF treatment, they will require some form of AHR treatment.

Many AHR international protocols exist—some examples of these are in vitro fertilization (IVF), gamete intrafallopian transfer (GIFT), pronuclear stage tubal transfer (PROST), tubal embryo transfer (TET), and zygote intrafallopian transfer (ZIFT) [2].

Assisted Human Reproduction (AHR) treatment is not regulated in Ireland, although it has been practiced since 1987 [1, 2].

Ireland is the only EU country without any form of AHR specific regulation. A draft Bill has been with the Irish government for three years but has not been introduced into law [3]. According to the Universal Declaration of Human Rights Article 16 states that the family is the natural and fundamental group unit of society and is entitled to protection by society and the State. The UDHR further provides for the right to marry and found a family “without any limitation due to race, nationality, or religion”. The UDHR in Article 25 also implicitly protects the unborn child by providing health services and social security for expectant mothers. Ireland as a member of the UN upholds the UDHR but has not implemented legislation around AHR regulation. Irish couples have travelled to other European countries to access these services in more regulated and cost-effective environments. This study aims to:

•Benchmark Ireland’s current status as regards AHR legislation and oversight.
•Review AHR legislation from other countries to identify best practice regulatory and patient oversight.

### Ireland and AHR

Infertility is defined as the failure to achieve a clinical pregnancy after 12 months or more of regular unprotected sexual intercourse. Infertility affects approximately 48.5 million couples worldwide (3% of the population), and in developed countries, infertility is diagnosed in 17–26 percent of reproductive age couples. Infertility is a worldwide health issue, with infertility rates increasing in recent years in all communities in both the developing and the developed world [4]. It is not only a medical condition, but it is also a social condition [5]. People live in a world where they have been conditioned to believe that they will all become parents one day, infertility carries considerable social implications [6, 7]. These implications not only affect the relationship of the infertile couple, but they also affect the couples' relationships with friends and family members [6–8].

#### History of AHR

The world's first baby conceived by in vitro fertilisation (IVF), a type of assisted reproductive technology (ART), was born in England in 1978 [9]. Since then, continuing improvements in clinical IVF were initiated in the early 1980s. Illustrative examples include, the development of ovarian stimulation regimens using different compounds during the follicular, midcycle and luteal phase, the improvement of embryo culture conditions, the development of transvaginal ultrasound, the cryopreservation of surplus embryos resulting in additional pregnancy chances, transport IVF, oocyte or embryo donation and improved embryo transfer techniques [9–11].

Experts, researchers, patients and religions have been clashing over infertility since its beginning [9, 11, 12]. Coupled with the speed of technological advancements and these clashes of beliefs have made it difficult to correctly legislate the processes [12]. Countries like Italy and Ireland, whose roots are grounded in the Catholic Church, struggle to introduce these laws as the Church has made its stand against ART throughout history [13]. In January 1976, the International Covenant on Economic, Social and Cultural Rights [14] put forward the right of everyone to enjoy the benefits of scientific progress. Access to ART and financial support is not universal often due to the country-specific rules and regulations governing ART. Each country has its own interpretation of ART rules, regulations and practices [15].

#### Ireland and AHR

Ireland has been practicing AHR since 1987. Currently, there are nine clinics that perform AHR treatments [16]. None of these clinics includes donor insemination and only one clinic offers donor eggs [15, 17]. The Irish Medical Council give the following guidance in relation to AHR in Sect. 47 of their Guide to Professional Conduct and Ethics for Registered Medical Practitioners. To summarise at a high level, the guidance states that in relation to assisted human reproduction that (1) IVF should only be used after thorough investigation has shown that no other treatment is effective (2) services should only be provided by qualified professionals, with accredited facilities, in line with international best practice, (3) for donor programmes there must be strong governance and records kept for donor traceability, (4) creation of human life for experimental purposes should not be engaged in nor should cloning [18]. The guide is not legally binding, and so the medical professionals in the AHR industry do not have to follow it [19] and not all AHR clinics register as medical practitioners [20]. The clinics are not obliged to submit details of treatments conducted in their facilities or publish any statistics on success rates. There is no authority in Ireland dedicated to AHR.

In 2000, the Irish Commission on Assisted Human Reproduction (CAHR) reported on potential approaches for the regulation of Irish AHR [17] but not all recommendations were unanimously supported hence the delay in the legislation. The report noted that genders and marital status should not be restrictive factors for AHR [17]. Articles 41 and 42 in The Irish Constitution describe the family as "superior to all positive law" and determines a woman's place as being "in the home" [17]. The government had set a date in 2018 to hold a referendum on the deletion of Article 41.2 but this referendum was postponed. Surrogacy is not addressed in Ireland [15]. Assisted reproductive technology has become a normalized part of reproductive medicine in many countries around the world. Access, however, is uneven and inconsistent, facilitated and restricted by such factors as affordability, social and moral acceptance or refusal and local cultures of medical practice. In Ireland, assisted reproductive technology has been available since 1987 but remains unregulated by legislation. This creates an uncertain and untenable legal circumstance given the contested issues related to constitutional protection of the right to life of the unborn and the indeterminate legal status of embryos in vitro. A decade has passed since the Commission on Assisted Human Reproduction in Ireland released its recommendations; the legislative vacuum leaves a potential legal limbo [15, 16] nor do the Medical Council guidelines discuss surrogacy or how embryos are disposed of.

Ireland is a signatory to Directive 2004/23/EC [17]. This Directive sets quality and safety standards for all treatments relating to human tissues or cells. The Health Products Regulatory Authority and the Commission on Product Safety and Quality Assurance have implemented the Directive via the regulation of laboratories and tissue management [16]. In 2015, the Children and Family Relationships Bill was introduced [19]. This Bill offers a definition to the term "parents" of children born through donor assisted human reproduction. There is no consideration in the Bill for sperm received from international donor banks on which Ireland relies heavily so heightens risks of unregulated sperm. According to the Health Review Board, the cost of a single IVF cycle in a private clinic in Ireland ranges from thousands of euro including travel and drug expenditure [20, 21]. Costs for AHR treatments vary as do charges for “add on” treatments. Most of these treatments have no proven safety or effectiveness data and are not regulated [22]. There are some means of claiming tax relief on these costs, and some health insurers in Ireland will assist with a portion of the costs. The Joint Committee on Health scrutinised the proposed Bill and issued a report in 2019 of its findings [23]. Several amendments to the Bill were suggested in the report. These amendments include removing age limits, removing the limitation on the number of embryos that can be implanted, including Pre-implantation genetic diagnosis (PGD) in any funding that will be made available and providing counselling services.

#### Adjunct treatments

Adjunct treatments can be offered to IVF patients [24]. These 'add-on' treatments are offered in all fertility clinics as they may improve their chances of a successful outcome to their treatment. There is a standard sequence of events for any new IVF technology that claim to improve live birth rates. First, the technology should be tested in an appropriate animal model, followed by testing in a clinical trial and finally in a randomised control trial. Only then is the treatment considered to be safe and effective. Further long term follow up studies, including paediatric studies, are completed to ensure the long-term safety of the treatment. Prospective parents undergoing IVF treatment generally pay large sums of money for treatment [2, 24]. The Human Fertilization and Embryology (HFEA) Act 1990 requires patients in the UK to be given all relevant information on any 'add-on' treatment, and they must give confirmed consent [25]. There is no consideration in Irelands', yet to be enacted, "Assisted Human Reproduction Bill" for 'add-on' treatments. UK Clinics must provide open and honest information on the evidence that surrounds an add-on treatment [22].

### Methodology

#### Literature review methodology

AHR is regulated in many other developed countries. These regulations differ from country to country, some are more stringent on fertility clinics whilst others are more advanced and consider reproductive cloning regulations. The regulations ensure the health and safety of women using AHR and the children that are born to them. The ethical use of AHR is also included in some of these regulations. The review of other counties regulations as part of this research was limited to the members of the Group of Twelve (G12) (which actually consists of 13 countries) [28], and these members regulations are reviewed in Sects. 3.2–3.14. Each of these countries has different AHR regulations, which are outlined in these aforementioned subsections.

The primary methodology employed in this research was literature review. A literature review can broadly be described as a more or less systematic way of collecting and synthesizing previous research [26]. The review protocol was planned as per the requirement of the research review was conducted by identifying, selecting and analysing the relevant papers and dissemination of the results in the form of descriptive results and thematic reporting [27]. The semi-systematic or narrative review approach was chosen for this research as it is designed for topics that have been conceptualized differently and studied by various groups of researchers within diverse disciplines and that hinder a full systematic review process [26, 27]. That is, to review every single article that could be relevant to the topic is simply not possible, so a different strategy must be developed. Thus, the semi-systematic review process requires more development and tailoring to the specific project research areas [27]. In this research the authors analyse specifically (1) Legislative and government regulatory documents and enacted law and (2) journal articles on legislative practices in different countries. Often, researchers need to develop their own standards and a detailed plan when utilising semi structured review to ensure the appropriate literature is accurately covered to be able to answer their research question and be transparent about the process [26]. There were many countries to be researched in terms of their legislative AHR practices, but only certain countries were under review.

Over a 6month period the authors searched articles in Google Scholar, SCOPUS, MEDLINE and Web of Science utilising search strings such as for example: “AHR LEGISLATION AND IRELAND”, “AHR LEGISLATION AND JAPAN” and “IVF AND JAPAN” and performed iterative subsequent search strings for each country under review. Articles found as a result of the search engine searches were then reviewed and scanned via their abstracts for relevance, selected if appropriate, read and cited if relevant to the research questions.

**Fig. 1:**
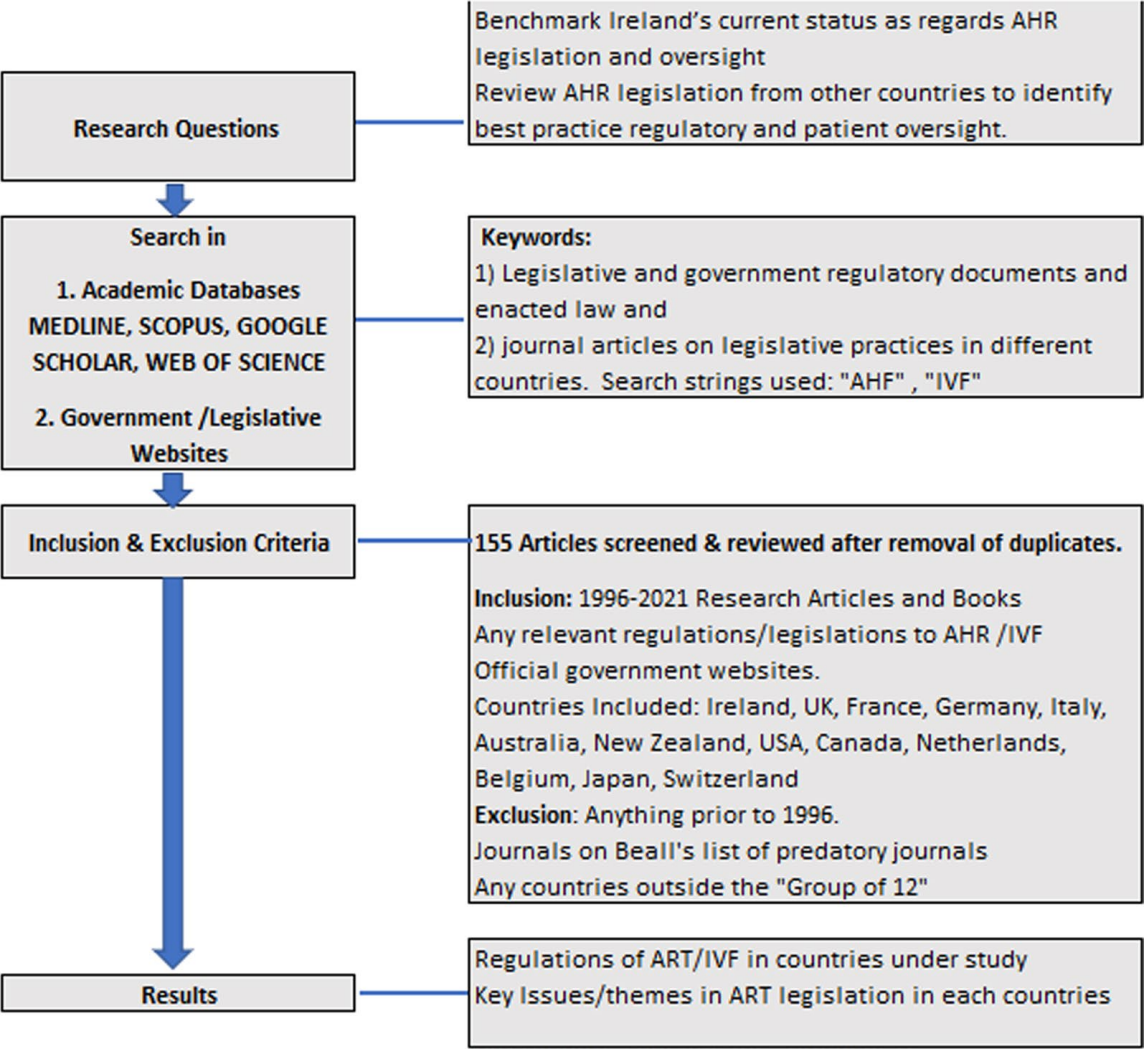
Methodology used for literature search

As legislation in a country or area changes constantly a literature review based on journal article review no matter how recent was not deemed to be accurate enough for this research as the articles may not be up to date with newly implemented legislation. Google and other search engines (Fig. 1) were used to access and identify country specific legislative websites and review regulatory information of different country specific regulatory authorities to overcome this issue. For example, the websites of the European Medicines Agency (EMA), American Food and Drugs Authority (FDA), Centre for Disease Control (CDC), Irish Health Products Regulatory authority (HPRA) to name a few were reviewed. Country specific legislative websites were also reviewed to review law and legislation and dates of enactments.

### Results—AHR in other developed countries

A number of themes within each country’s legislation in relation to AHR was explored and discussed. The primary themes were: the Legal frameworks and public/government support for ART. Within the legal framework category, the legal age limit for ART access, the legal limits for ART third part donations and preservation of fertility preservation. Within the public and government support category the supports that are publicly available and types of services supported are discussed.

#### Australia

AHR in Australia is regulated at a federal and state level. The "Prohibition of Human Cloning for Reproduction and the Regulation of Human Embryo Research Amendment Act 2006" is the primary federal law [28, 29]. Assisted reproduction clinics in Australia must follow the "Ethical Guidelines on the Use of Assisted Reproductive Technology in Clinical Practice and Research" published by the National Health and Medical Research Council (NHMRC) [30]. These guidelines must be followed in order for the clinic to be accredited by the Reproductive Technology Accreditation Committee (RTAC). The guidelines limit the number of embryos that can be created, the recording of results and surrogacy. The clinics are audited against the Australian and New Zealand Code of Practice. The Human Assisted Reproductive Technology Act (HART) in 2004 is the equivalent governing Act in New Zealand [31]. In 2010, Victoria further expanded its legislation and included an addendum to birth certificates for donor conceived children born after 2010 [32]. Under this addendum, donor conceived children will be notified as an adult on the application for a birth certificate that they were conceived via donor AHR and will be able to apply for information about their donor from the registry.

#### Belgium

In 2002 and 2007, Belgium introduced its key AHR laws, the “Law on Research into Embryos In Vitro 2002” and the “Law on Medically Assisted Reproduction and the Disposition of Supernumerary Embryos and Gametes 2007” [33]. The laws include limitations on cloning, embryo research and hybrid embryo creation. The law regulates four different sections of AHR. First, the disposition of embryos; second, the donation of gametes and embryos; third, the specific applications of assisted reproduction such as post-mortem reproduction and lastly, the use of preimplantation genetic diagnosis for HLA typing. The law also contains requirements for a contract, the maximum storage period and the procedure for when a patient is refused treatment [34]. Since 2003, AHR treatments have been covered by the National Health Plan in Belgium. Six cycles of AHR are covered for women under the age of 42. Women aged over 43 years are not eligible for the cover. There are stringent limitations on the treatments, including the number of embryos that can be transferred in one cycle [35].

#### Canada

AHR in Canada is legislated under the Assisted Human Reproduction Act (2004) [36]. The Assisted Human Reproduction Agency of Canada (AHRA) is responsible for enforcing the Act and the regulations [37]. Cloning is prohibited under the Act, as is research into cloning. The legislation also prohibits the selection of sex, hybrid embryos, commercial surrogacy and trading of human eggs, sperm and embryos. There is a series of principles in the regulations; included in these principles is the discouragement of discrimination against same-sex couples and single mothers. There is also a principle discouraging the use of AHR for commercial gains. In 2016, the Act was strengthened [38]. Health Canada introduced new regulations within the Act to protect the safety of donor sperm and ova, reimbursement and administration and enforcement, which were published in 2019. There are 10 Canadian provinces, but only four provinces provide financial assistance for AHR [39].

#### France

AHR clinics in France are regulated by the French Biomedicine Agency. The critical laws in France are the "Bioethics Law No. 2004–800 (2004) " and the "Law on the Donation and Use of Elements and Products of the Human Body, Medically Assisted Procreation, and Prenatal Diagnosis, No. 94-654 (1994)" [40]. Cloning is prohibited under these laws, as is the creation of embryos for research. Sex selection and surrogacy are also prohibited [41]. France is one of the six countries in the world where ART is fully covered by public funding; the provision was not extended to lesbian couples, single women and women of a certain age [42]. Complete coverage of financial costs is provided by the National Health Plan in France. France's new IVF law aims to extend assisted reproduction technology (ART) coverage to same-sex couples and single women, making treatment free under the country's national healthcare system [43].

#### Germany

In 1990, Germany introduced the Embryo Protection Act (ESchG) [44] and ensured that the embryo was preserved and introduced penalties if clinics are non-compliant. The regulation restricted AHR for use only in achieving a successful pregnancy and not for any other purpose. The 'rule of three' in the legislation limits the number of embryos that can be transferred in a single cycle to three. Misuse of AHR as defined by the legislation include egg cell donation, surrogacy and the use of sperms and eggs from a deceased party [45]. The donation of sperm cells and the donation of embryos are not mentioned in the framework.

The "Federal Embryo Protection Law 1990", the "Adoption Brokerage Law 2006" and the "Guideline of the German Federal Medical Chamber 2006" are the main German laws. Cloning is prohibited under these laws, as is the creation of embryos for research and the creation of hybrid embryos [46]. Preimplantation genetic diagnosis, sex selection, embryo selection and surrogacy are also prohibited. There is still a need for a law in Germany that regulates all aspects of AHR. Introducing this into law would spark a series of public debates on the status of embryos and when human life begins [45]. The partial regulation leaves considerable gaps in AHR treatments; one such gap is the handling of the embryos not implanted due to the 'rule of three'. The "Tissue Act" was introduced in 2007 to establish the standards of quality and safety for egg cells, sperm cells, oocytes, and embryos [47]. In 2002, the "Stem Cell Act" was introduced to protect human gametes and outline the conditions for research into embryonic stem cells. German physicians have highlighted that due to the legal restrictions, they are not offering the most advanced treatments to their patients. One such restriction, the embryo selection restriction, has resulted in patients requesting to be implanted with a maximum of three embryos. This has resulted in a rise in unwanted multiple pregnancies [48, 49].

AHR funding in Germany statutory health insurances was restricted to 50% in 2004, whereas prior to this, the treatments were 100% funded [50]. Insurance benefits for assisted reproduction are regulated in a special paragraph in the Code of Social Law [50, 51]. This paragraph details the limitations covered; there are age limits (female age 25–39, male age 25–49), as well as other limitations (couples must be married, HIV negative and must not need a sperm donor). There is an ongoing public debate on the funding cut. However, when not considered in isolation and included in the list of other health care services requiring funding, AHR ranks last out of 17 other kinds of funded healthcare in Germany [50].

#### Italy

The “Medically Assisted Procreation Law” was introduced in Italy in 2004 [12]. This law (law40/2004) opened with the statement that AHR is only acceptable to solve reproductive problems. Under this law, a national register of authorised AHR clinics was created. Strict standards were introduced for the clinics registered. Data is collected from the clinics by the register, and they are monitored to ensure they meet safety and effectiveness standards. The law demands that quality audits on the operators' professionalism, the adequacy of the equipment and applied technologies are carried out on the clinics regularly. Under the law, a maximum of three eggs can be fertilised and transferred per cycle; as discussed previously, it was the law at one stage in Italy to implant all viable embryos [52]. This, as with other countries legislation, has resulted in an increase in unwanted multiple pregnancies.

The law was widely criticised on its introduction [12, 52]. More than 30 challenges to various aspects of the law were taken to Italian Courts. The lack of access to preimplantation genetic diagnosis (PGD) and donor insemination were the main aspects of the law criticised as well as the criteria for accessing AHR treatments [53]. In April 2014, the Constitutional Court ruled that gamete donation techniques were immediately applicable within the regulatory framework currently in force [54].

Though this change and other changes have been made to the law over time, PGD is still prohibited, but access has been granted to some couples on a case-by-case basis. Restrictions are also still in place for access to treatments. These restrictions reinforce the idea of the "appropriate" family being heterosexual parents who are married or in a stable relationship, and thus, access is restricted to heterosexual couples who live together and are of reproductive age. Also, the couple must first have been provided with the option of adoption; this is a unique restriction. Key issues remain unresolved in Italy, such as the withdrawal of consent to the procedure until the time the oocyte is inseminated and the further liberalisation of the basic criteria to access AHR (single women, lesbian and gay couples) [29]. The legal framework in Italy has been described as one of the strictest in the world and at the most conservative end of Europe in the world [52]. The Italian National Health System covers the majority of AHR treatments, although there are minor regional differences. [9, 55].

#### Japan

Today Japan is one of the largest users of AHR worldwide; it has the highest number of registered fertility clinics [56]. This fact can be accredited to the culture in Japan where women are supposed to have children and carry on the family [54, 56, 57]. However, the only law governing AHR in Japan was introduced in 2001. The "Law Concerning Regulation Relating to Human Cloning Techniques and Other Similar Techniques" prohibits cloning (with the exception of research cloning), gene modification and hybrid embryos. The Japan Society of Obstetrics and Gynaecology started an online cycle‐based assisted reproductive technology (ART) registry system in 2007 [56]. This report presents the characteristics and treatment outcomes of ART registered for the cycles practiced during 2016 [57]. All other AHR treatments are regulated by guidelines created by the Japan Society of Obstetrics and Gynaecology (JSOG) [58]. These guidelines alone serve as the legal framework for AHR in Japan, despite having no legal enforcement. These guidelines prohibit the use of donor eggs and embryos and also prohibit surrogacy [59]. In 2007, JSOG started an online registry system for AHR [60]. Japanese couples IVF rates have been increasing in recent years [59] and requires some form of AHR [53, 58]. More of these couples are travelling abroad for treatments due to the prohibitions in the guidelines [61].

In 2004, the Japanese government introduced a subsidy programme to provide financial support to infertile couples [62]. This was intended to encourage the use of AHR. In 2016, the government imposed an age limit on female patients granted access to the programme and reduced the number of IVF cycles to be funded to 6 for women under 40 and 4 for women aged 40–42. Reproductive tourism is fast becoming a commercialised part of Japanese life as couples travel to nearby Asian countries for cheaper treatments and access to prohibited Japanese treatments [61, 63, 64].

#### Netherlands

The laws for AHR in the Netherlands are the "Act Containing Rules Relating to the Use of Gamete and Embryos (Embryos Act) 2002" and the "Commercial Surrogacy Act 1993" [65]. There are also laws on the safety and quality of human tissues which can be applied to AHR. In 1998 the Netherlands introduced the "Act on In Vitro Fertilization", and in 2002, the "Law on data from donors for artificial reproduction" was introduced [33]. Research on embryos, the development of an embryo outside the human body for more than 14 days, cloning, gene modification, hybrid embryos, sex selection, embryo donation and gamete donation is prohibited under the embryo act. Commercial surrogacy is prohibited under the "Commercial Surrogacy Act" [34]. PGD is only legal for couples who have a prior history of a serious genetic disease. There are only commercial clinics in the Netherlands; there are no private AHR clinics. There is an accreditation system in place for clinics. Accreditation involves a license and inspection by National Health Inspectorate. The standard health insurance package in the Netherlands includes IVF, which everyone living or working in the Netherlands is obliged to have. The Dutch government decide the amount of cover to be provided to IVF treatments; this can change annually. There are limitations to the cover; the woman must be under 42 years old and must have failed to achieve a pregnancy for a number of years.

#### Spain

There are two laws in Spain that govern AHR. Law 35/1988 "assisted reproduction techniques" which was modified in 2003 to Law 45/2003 and Law 14/2006 "Law on Assisted Human Reproduction Techniques". Cloning, transferring more than three embryos per cycle, embryo experimentation, gene modification, sex selection (except when avoiding disease) and non-medical PGD is prohibited. Donor gametes are permitted in Spain, but only six children are permissible to be born from one donor. Law 14/2006 includes a list of all current AHR treatments and techniques but allows experimentation with new techniques in order to advance AHR treatments. Physicians are expected to enact self-control in experimenting with these techniques. There are few limitations on access to AHR in Spain. Marital status, sexual orientation and post-mortem status are not limiting factors for access. Treatment is denied when the health of the women or the child is at risk. Law 14/2006 protects the leftover embryos from AHR. The law encourages the donation of healthy embryos for research purposes. Spain does not acknowledge surrogacy [2].

The committee in Spain for AHR issues is the National Commission on Human Reproduction Assistance. There is an accreditation system in a place licensed by competent local authorities. The national registry for AHR is organised by The Spanish Fertility Society. Clinics have an obligation to report local registries for AHR treatments. Full coverage is provided to patients who attend public AHR clinics. Patients are reimbursed. Some treatments, however, such as PGD, are only performed in private AHR clinics.

#### Sweden

Sweden has two laws governing AHR, the "Act on Ethics Review of Research Involving Humans, Law No. 460 (2003)" and the "Genetic Integrity Act, Law No. 351 (2006)". The legislation has been described as moderate; it is neither very restrictive nor very liberal. Sweden has both public and private clinics. Cloning, PGD (for non-medical purposes), surrogacy and gene modification is prohibited. Gamete donation is acceptable only in a university hospital, and embryo donation is prohibited. Only one embryo is allowed to be transferred per cycle. Two embryos are allowed in older women. Cryopreservation is al-lowed for up to 5 years.

There are age limits in Sweden for accessing treatments. The limit for women is between 25 to 40 years old, increased to 45 years old if using frozen embryos, and 56 years old for men. There is no limitation due to marital or sexual status. Sweden is a secular country; there is no controversy in relation to gamete donation, same-sex families, single-parent households as there are in many Catholic countries [66]. Controversies in Sweden mainly concern how the technologies are practised, such as access to treatment, donor anonymity, commercialisation of surrogacy, commercialisation of eggs, commercialisation of gametes, and AHR conceived children's rights [67].

The Genetic Integrity Act (Swedish Code of Statute 2006:351) stipulates that married couples, registered partners, cohabiting partners and single women can undergo insemination or in vitro fertilisation within the Swedish health care system [68]. Financial coverage is provided. Access to financial coverage is only provided to those who are married or are in a stable relationship.

#### Switzerland

Switzerland has two laws governing AHR. The “Federal Law on Medically Assisted Reproduction (1998)” and the “Federal Act on Research Involving Embryonic Stem Cells (2003), and the Federal Law on Medically Assisted Reproduction (2004)” [69–71]. These laws prohibit cloning, donation of eggs and embryos, embryo creation solely for research, hybrid embryos, gene modification, PGD, eSET, sex selection (unless for medical purposes) and surrogacy. The number of embryos that can be transferred in one cycle is limited to three. The laws allow cryopreservation of gametes and embryos, but they must be destroyed after five years. All the data on AHR in Switzerland is collected by the profession-al community in Switzerland, which voluntarily set up an organisation to provide transparency into the industry [72]. Current legislation has limited the advancement of AHR in Switzerland. There have been attempts to modify the laws to include PGD and to extend the number of embryos that can be cultured to eight, but these attempts have failed [73]. Accessibility to AHR is legally restricted to heterosexual couples. There is no funding for patients accessing AHR in Switzerland. There is an option for patients to apply for a tax deduction post-treatment, but this depends on the Canton of residence [2].

#### United Kingdom

AHR has been regulated in the United Kingdom (UK) for over 25 years. There are three main laws governing AHR in the UK; the “Surrogacy Arrangement Act” introduced in 1985, the “Human Embryology & Fertilisation Act” introduced in 1990 and the “Human Reproductive Cloning Act” in 2001. In 1991 the Human Fertilisation and Embryology Authority (HFEA) was founded [25]. Clinics require a license from the HFEA in order to operate. The HFEA license and inspect clinics. An initial inspection is carried out before the issuing of a license. After this initial inspection, clinics undergo regular unannounced inspections and scheduled inspections. Patient records, including consent forms, are inspected by the HFEA in accordance with the HFEA's code of practice and with the 1990 Act (amended in 2008). The HFEA even interview patients. A negative report from the HFEA can result in the revoking of a clinics license [74].

All treatment and results must be reported to the HFEA. Thus, there is a large dataset in the UK of AHR results. The HFEA specify how these results should be reported, and therefore each clinic can be adequately compared. The data has consistently shown that there is minimal variation between clinics success rates. Patient's characteristics have shown to have more of an impact on success rate than the clinic's practices [75].

The AHR laws in the UK prohibit cloning, cross-species embryo transfer, gene modification. Sex selection (for non-medical purposes), surrogacy, egg donations, sperm donation and the development of an embryo outside the human body for more than 14 days. The number of embryos that can be transferred in a single cycle is limited to two for women under 40 years old and increases to three for women over 40 [76]. Embryo cryopreservation is permissible, but the embryos must be destroyed after ten years [77]. New laws have been passed in the UK removing the anonymity of gamete donors. Once over 18 years old, a child conceived by egg, sperm or embryo donations now has the right to information about their genetic parents [78].

The HFEA has no authority over the prices that clinics charge or their marketing of adjunct treatments. The 1990 Act states that the concerns regarding practices at clinics can be addressed to the regulator. The Act states that it is the responsibility of the clinic to ensure 'that suitable practices are used in the course of the activities.

IVF is free in the UK for women who are under forty [78] but not for over forties which has led to UK women over 40 travelling abroad for access to cheaper AHR treatments.

#### United States of America

AHR in the United States of America (USA) is regulated at a federal and state level. In 1992, the “Fertility Clinic Success Rate and Certification Act” was introduced to legislate AHR at a federal level [79]. The Centers for Disease Control and Prevention (CDC), the Food and Drug Administration (FDA) and the Centers for Medicare and Medicaid Services are responsible for enforcing the Act [79–81]. Under the Act, clinics are required to report success rates to the CDC. However, this reporting has been misleading in some cases as the data has been reported in such a way to inflate success rates in some clinics [82–84]. Some clinics do not report their data to the CDC. The CDC is not fully aware of every practicing clinic and requests patients to notify them of any clinic that is not on their list of centres [81, 85]. At the individual state level, the regulations for AHR vary. Some states have very limited regulations on AHR, while others are more comprehensive [85, 86]. Cloning is prohibited in some states; surrogacy is prohibited in others. There are a number of states that require private insurance for the funding of AHR [87, 88]. Only one state, Pennsylvania, inspects and provides extensive regulation for AHR clinics and practices [89]. The 1992 Act provides states with a model embryology laboratory certification process; implementation of this model is not mandatory. Thirteen states have no regulations for AHR. The FDA is limited in its governance of AHR. The code of Federal Regulations-21 CFR Part 1271 sets standards for human tissue and tissue-based products but does not cover reproductive tissue [89]. There is, therefore, a wide range of variations in sperm banks, genetic screen and other reproductive tissue treatments across clinics [90]. The “Good Tissue Practice” regulations contain minimal sections relatable to reproductive establishments. Additionally, there are professional guidelines and good practice protocols developed by The American Society of Reproductive Medicine (ASRM) and the Society for Assisted Reproductive Technology (SART) that some clinics follow [91–93]. The guidelines include limitations on age and embryo transfer numbers (one for women under 35 and no more than two per cycle).

### Discussion

The literature review and review of legislation and practices across many global countries identified how AHR is regulated in Ireland versus other countries. Ireland is a conversative county compared to other jurisdictions and in some areas such as IVF legislation and is lagging behind other countries.

Different themes arose in different countries in relation to the type and scope of AHR legislation and the provisions therein as well as the type of procedures, services and treatments that can be accessed, the level of government support for those services, and the rules around who can access.

The main legal differences in AHR between countries relate to cryopreservation, PGD, gamete donation, surrogacy, limitations for access and funding. The legal standpoint on embryo research is also a key difference and one that determines how advanced the countries approaches and technologies are in relation to AHR treatments. There are some common parts of the legislation, in particular for the EU countries due to the introduction of the EU Tissue & Cell Directives [94].

Due to differences in social and religious beliefs and the pace of advancement of AHR technology—each country does things differently from a regulatory viewpoint. The European Society of Human Reproduction and Embryology (ESHRE) Task Force on Ethics and Law however has determined the key aspects in AHR for regulating access and impartiality. The task force also denotes that the treatments should at least be partially funded [95].

There is no legislation in Ireland specific to AHR that service provider clinics are obliged to follow. The HPRA play a role in AHR as the Competent Authority for European and national legislation governing tissues and cells and they authorise and inspect all clinics performing IVF in Ireland. The primary legislation is the European Directive 2004/23/EC, which sets standards of quality and safety for the donation, processing, preservation, storage and distribution of human tissues and cells. The HPRA remit is limited specifically to matters related to the quality and safety of the tissues and cells.

There is no visibility to provide insight or comparison into the different fertility clinics in Ireland. These clinics report their success rates in different ways and can offer add-on treatments without any scientific proof of their effectiveness, as is happening in the USA and mentioned previously. On analysis of the new Irish proposed legislation by the Joint Committee on Health in 2019—some changes which may bring Ireland more in line with European county specific practices were proposed. These include removing age limits, removing the limitation on the number of embryos that can be implanted, including PGD in any funding that will be made available and providing counselling services. In addition to these recommendations, funding integrated into legislation as in other countries has been put forward as a best practice aid.

The new proposed Irish legislation will introduce a dedicated, competent authority—the assisted human reproduction regulatory authority (AHRRA) which will provide regulatory oversight of Irish AHR. The objective of the AHRRA is to protect, promote and, as far as practicable, ensure the health and wellbeing of children born as a result of assisted human reproduction, the intending parents, and other persons involved in the process. Similar to the UK system, the AHRRA will grant licences and will have the power to revoke these licenses if clinics are not complying with the conditions outlined in the Act. The AHRRA will publish and maintain codes of practice giving guidance for the proper conduct of activities.

The review of other countries regulations was limited to the members of the Group of Twelve (G12). Each of these countries has different AHR regulations with different approaches and each has its advantages and disadvantages from both a legislative and patient experience viewpoint. However, the legislation in the United Kingdom, if adopted in Ireland, would adequately close the legislative gaps that have been identified in this research.

IVF is free in the UK for women who are under forty. The HFEA has no authority over the prices that clinics charge or their marketing of adjunct treatments. Best practice in many countries is to have set price guidelines for the treatments, with a financial support system in place. Linking IVF to health insurance is also favoured by many countries Although some health insurance companies in Ireland offer IVF cover, not all treatments and clinics may be covered. The financial support system in the Netherlands has a standard obligatory mandated health insurance package that includes IVF.

### Conclusions

Ultimately it's country specific domestic politics aligned with the countries affiliations and membership with other global and political bodies and in some cases their religious ethos that have a role in adoption policy of any international regulations, policies and treaties. This research has discussed that there is no legislation in Ireland specific to AHR that regulates service provider clinics. The findings from this research will be useful to medical practitioners who wish to advise patients on AHR and members of the public who wish to avail of AHR services in terms of providing a reference and summary for them. As a benchmark and best practice model, the authors proposed a hybrid system similar to the UK’s and Netherlands AHR regulatory system. The UK is the best model in terms of organisation, practice, regulation and surveillance. The Netherlands is presented as a best model in terms of a financial support system. The limitations of this study would be that it was a solely literature-based research. There is an opportunity for qualitative and quantitative study in terms of patients experiences of the Irish AHR system, which are being investigated by the authors.

## Data Availability

Not applicable.

## Abbreviations

AHR: Assistant human reproduction
IVF: In-vitro fertilisation
ART: Assisted reproductive technology
PGD: Pre-implantation genetic diagnosis
HFEA: Human Fertilisation and Embryology Authority
G12: Group of twelve
ESHRE: European Society of Human Reproduction and Embryology
AHHRA: Assisted Human Reproduction Regulatory Authority
ASRM: American Society of Reproductive Medicine
SART: Society for Assisted Reproductive Technology (SART)
NHMRC: National Health and Medical Research Council
HART: Human Assisted Reproduction Technology
EMA: European Medicines Agency
FDA: Food and Drugs Authority
CDC: Centre for Disease Control
HPRA: Health Products Regulatory authority (HPRA Ireland)
EU: European Union
UK: United Kingdom
USA: United States of America
AHRA: Assisted Human Reproduction Agency of Canada
HFEA: Human fertilization and embryology
